# Levels of Self-representation and Their Sociocognitive Correlates in Late-Diagnosed Autistic Adults

**DOI:** 10.1007/s10803-021-05251-x

**Published:** 2021-08-30

**Authors:** R. L. Moseley, C. H. Liu, N. J. Gregory, P. Smith, S. Baron-Cohen, J. Sui

**Affiliations:** 1 Department of Psychology, Bournemouth University, Bournemouth University, Talbot Campus, Fern Barrow, Poole, Dorset, BH12 5BB UK; 2 Autism Research Centre, Department of Psychiatry, University of Cambridge, Cambridge, UK; 3 Present Address: School of Psychology, University of Aberdeen, Aberdeenshire, UK

**Keywords:** Self-bias, Self-representation, Social cognition, Mentalizing, Loneliness

## Abstract

The cognitive representation of *oneself* is central to other sociocognitive processes, including relations with others. It is reflected in faster, more accurate processing of self-relevant information, a “self-prioritisation effect” (SPE) which is inconsistent across studies in autism. Across two tasks with autistic and non-autistic participants, we explored the SPE and its relationship to autistic traits, mentalizing ability and loneliness. A SPE was intact in both groups, but together the two tasks suggested a reduced tendency of late-diagnosed autistic participants to differentiate between familiar and unfamiliar others and greater ease disengaging from the self-concept. Correlations too revealed a complex picture, which we attempt to explore and disentangle with reference to the inconsistency across self-processing studies in autism, highlighting implications for future research.

**Supplementary Information:**

The online version contains supplementary material available at 10.1007/s10803-021-05251-x.

Autism spectrum conditions (ASC) were originally described as conditions of ‘extreme aloneness’ (Kanner, 1943), and difficulties in developing and maintaining social, romantic and professional relationships blight the lives of autistic people (Causton-Theoharis et al., 2009; Deckers et al., 2017; Hendricks, 2010; Mazurek, 2014; Strunz et al., 2017). These difficulties have been commonly attributed to differences in theory of mind (ToM) or ‘mentalizing’, and its neural substrates (Baron-Cohen, 2009; Fishman et al., 2014; Murray et al., 2017; Rosenblau et al., 2015). Whilst the question as to how autistic people relate to others has always sat at the heart of autism research, only more recently have researchers considered relationships between other-processing and representation and cognition around the *self* in autism. As the interpersonal difficulties of autistic people have indeed been linked to heightened emotional arousal in oneself (Gu et al., 2015), differences in self-representation may in fact be a propitious window to understanding other features of autism.

In non-autistic (NA) people, self-representation impacts on a range of cognitive and emotional processes, and consequently influences social interaction (Sui & Gu, 2017; Sui & Humphreys, 2015a). Objects belonging or related to oneself are imbued with the power to ‘highjack’ attentional resources (Humphreys & Sui, 2016) and are preferentially recalled (Turk et al., 2011). When previously-neutral linguistic stimuli or geometric shapes are instilled with self-relevance, recall and processing of these stimuli is likewise facilitated (Kelley et al., 2002; Leshikar & Duarte, 2014; Macrae et al., 2018; Sui et al., 2012, 2013a, 2013b). The self functions as an ‘anchor’ in decision-making and an ‘integrative glue’ in memory (Sui & Humphreys, 2015a), above and beyond effects of familiarity and reward-bias (Sui & Humphreys, 2015b). At brain level, self-referential processing involves multiple interconnected networks, including regions involved in mentalizing about the emotional and mental states of oneself *and* others (Steinbeis, 2016; Sui & Gu, 2017).

The influence of different levels of self-representation on social and cognitive processes, and the overlapping substrates for self- and other-mentalizing, raises the possibility that through examining *self* representation, we might better understand cognition and social relationships with other people. Early theorists understood ‘extreme egocentrism’ to exist hand-in-hand with the quintessential ‘aloneness’ of autistic people (Lombardo & Baron-Cohen, 2010). In an expansive review, these authors note developmental delays in processes linked to having a concept of self (such as orienting to one’s name, understanding pronouns, and developing joint attention), along with enduring differences, in adulthood, in the ability to monitor and differentiate one’s own intentions, emotions and thought processes from those of another (Lombardo & Baron-Cohen, 2010). These authors suggest these differences might originate in reduced strength of distinction between self and other, a view supported by differences in structural and functional connectivity within relevant circuits (Abbott et al., 2016; Burrows et al., 2016; de Lacy et al., 2017) and by differences in brain activity during processing of self-relevant information (Lombardo et al., 2010).

To better understand self-representation in autism, researchers have examined the mnemonic advantages of self-relevant stimuli in autistic people without intellectual disability. Studies in both children and adults have reported reduced prioritization of self-owned items (Grisdale et al., 2014; Wuyun et al., 2020), and self-relevant adjectives (Burrows et al., 2017; Henderson et al., 2009; Lombardo et al., 2007)—but this latter finding was not corroborated in a recent large-scale study (Lind et al., 2020). Likewise, the diminishment of the mnemonic SPE in accordance with increasing autistic traits in autistic individuals (Grisdale et al., 2014; Henderson et al., 2009; Lombardo et al., 2007), has not always been replicated (Lind et al., 2020). A role for the self in social cognition predicts relationships between reduced SPE and mentalizing difficulties, but these are likewise somewhat tenuous, present in some studies (Lombardo et al., 2007) but not in those which controlled for age, verbal IQ and diagnostic status (Henderson et al., 2009).

These contrasting findings leave the nature of the SPE in autism still uncertain, and this may be in part because researchers have tended to mistakenly conceptualize the self as a unitary cognitive structure and, with specific tasks, focus on one component alone. In fact, reflecting the multidimensional nature of the self-concept, self-biases manifest differently across cognitive domains (Nijhof et al., 2020). Many of the aforementioned tasks require a higher-level kind of conscious processing, explicitly evaluating adjectives with reference to an abstract notion of the self—such that higher-level difficulties with self-understanding may obfuscate more fundamental differences in self-prioritisation (Gillespie-Smith et al., 2018). Accordingly, Williams et al. (2018) utilised the shape-matching task of Sui et al. (2012) to examine lower-level responses to self-relevant information in the perceptual domain, understudied with reference to the SPE in autism. In two experiments, they reported a similar SPE in autistic adults and a non-autistic comparison group, and a lack of relationships between the SPE and two measures of autistic features and two measures of mentalizing ability. On the apparent normality of the SPE in perception for autistic people and the absence of these relationships, the authors suggested that differences in self-representation, in autism, might be restricted to the mnemonic domain, where tasks require more complex judgements of second-order representation (“Does this trait apply to *me*?”). Grisdale et al. (2014) likewise suggested that different levels of self-representation might be ‘blocked off’ from influencing one another across cognitive domains—such that, in their study, conscious, deliberate awareness of the ‘psychological self’ in a self-report measure was belied by the absence of SPE in the memory task.

As the Williams et al. study involved a small autistic sample (n = 22), our study aimed, firstly, to test the replicability of their findings concerning perceptual SPE in autistic individuals and relationships between the SPE and autistic traits. If the SPE were indeed intact at the perceptual level, autistic and NA people alike were expected to affirm relationships between stimuli with greater speed and accuracy when stimuli were associated with their self-concept. In extension to the previous work, however, we attempted a broader scrutiny of two levels or components of self-representation: the aforementioned SPE effect at the perceptual level, and a higher-level conscious conceptual representation of the self in relation to others, namely a friend and a stranger. Through examining both low- and high-level self- and other-referential processes in one autistic sample, our first goal was thus to obtain a clearer perspective on the features of both, and to examine whether the typical hierarchy, where self is first but friends are prioritized over strangers (Sui & Gu, 2017), was present, because in addition to distinguishing between the self and others, a differentiation between familiar and unfamiliar is a crucial ability for development and ageing (Carver et al., 2006; Sui & Humphreys, 2017). Given the typical dissatisfaction that autistic people often report with their relationships (Moseley & Sui, 2019), this uninvestigated friend-prioritization effect (FPE) could not be presumed.

Our second aim was to examine relationships between differences in self- and friend-representation and the social difficulties associated with autism. These being archetypally linked with differences or difficulties in ToM, the interconnectedness of self- and other-representation could be a means through which differences in self-representation could affect social outcomes. Though the use of tools such as the Reading the Mind in the Eyes Test (RMET) in previous research on the SPE is thus motivated, the extent to which performance on ‘ToM tests’ translates to real-life social outcomes has been queried (Bottema-Beutel et al., 2019; Murphy & Lilienfeld, 2019; Schneider et al., 2019). As such, whilst we attempted to clarify the previously ambiguous relationship between RMET scores and self-representation in autistic people, we also examined relationships between both higher- and lower-order aspects of self-representation and a more direct measure of social difficulties, the UCLA Loneliness Scale (Russell, 1996). Hypothesising a key role for self-representation in other aspects of cognition and in social relationships, we predicted that in a larger autistic sample than that of Williams et al., relationships might be seen between the extent of the SPE, the FPE, mentalizing abilities (RMET) and loneliness.

## Method

### Participants

Autistic participants (n = 120; average age: 40.7 years [SD: 13.4]) were recruited from the ranks of individuals who had participated in previous studies by the authors (citations to be added), and through social media (Facebook groups). It so happened that most of our participants were diagnosed as adults (mean age: 35.6 years [SD: 14.2]). Diagnoses could not be independently verified, but participants reported the date, location and precise diagnosis given, along with any additional diagnoses (8% reported comorbid ADHD/ADD; 13.3% had dyslexia, dyspraxia or a specific learning difficulty). It was not possible to obtain IQ measures, but it is highly likely that participants were in the average to high range: all participants were qualified to GCSE level, and 55% were qualified to at least degree level. Just under half of the participants (49.2%) were employed in some kind of paid work.

Forty-four non-autistic (NA) participants (average age: 26.7 [SD: 8.6]) were recruited through social media and from the student cohort at the first author’s institution. They were screened for autism and ADHD, but two had dyslexia and nine reported an autistic first-degree relative (parent, sibling or child). Psychiatric illnesses were substantially more common in the autistic group (56% and 50% reported mood and anxiety disorders respectively, as compared to 20% and 25% of NA participants), 47% of whom were taking psychotropic medication (9% in the NA group). The majority of participants were British (65% autistic, 80% NA), followed by American (10% autistic, 7% NA), European (8% autistic, 9% NA), Canadian (3% autistic, 2% NA); a further 3% of autistic participants were from Australia or New Zealand, 0.8% from South America, and 10% declined to answer.

### Materials and Procedure

All procedures were pre-approved by the Faculty Ethics Committee at the first author’s institution. This investigation of self-referential processing employed two tasks, illuminating the representation of an individual’s self-concept at different levels.

**Fig. 1:**
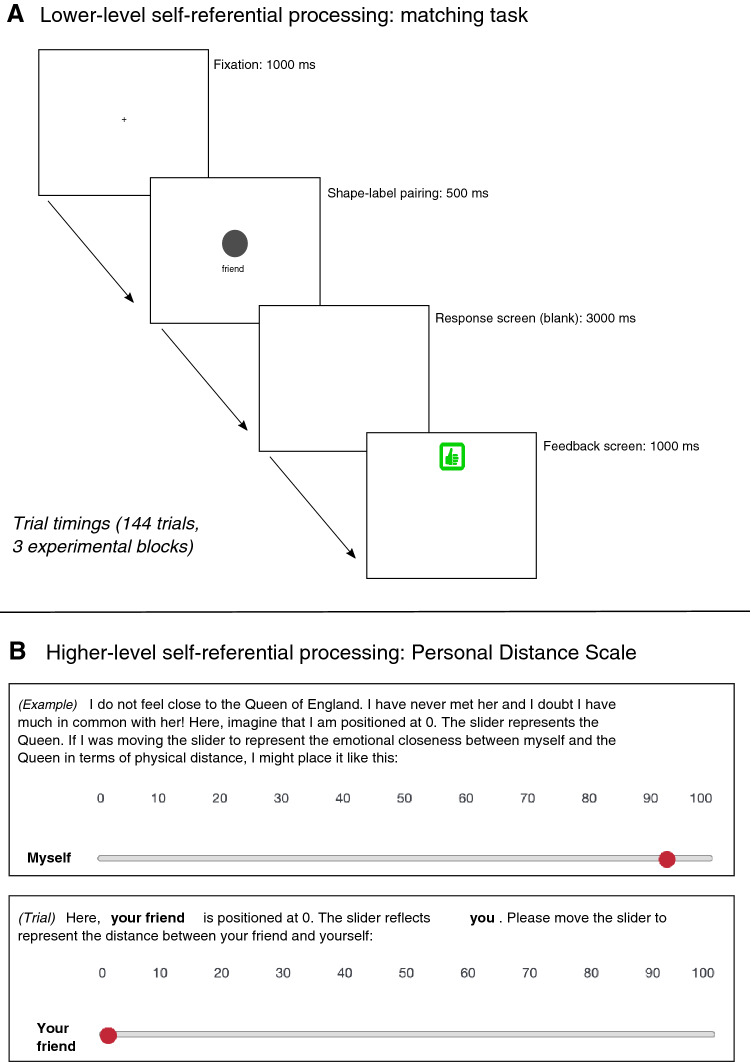
Schematic for the matching task and Personal Distance Scale

To investigate self-referential processing at the lower, perceptual level as evinced in the SPE, participants completed a matching task adapted from Sui et al. (2012) for online delivery via Tatool (von Bastian et al., 2013). In this task, which was piloted on independent NA participants prior to the study (see Supplementary Materials 1 for details), participants learnt associative relationships between shapes (circles, squares and triangles) and person labels (‘yourself’, ‘friend’ and ‘stranger’). In experimental trials, participants saw the same shapes and labels paired pseudo-randomly and, as quickly as possible, affirmed or negated with a keyboard response whether the labels attached to the shapes matched the pairings they had learnt (Fig. 1, Part A). The self-bias effect is independent of the shape associated with ‘yourself’, ‘you’ or ‘me’ (Sui et al., 2012), but shape-label pairings were counterbalanced across participants in three otherwise-identical versions of the task. (Once we had checked for main effects or interactions of ‘task version’, the counterbalancing was deemed effective and this factor was dropped from analysis). Each block contained 48 trials: 24 matching trials (8 for each of the three shape-person pairings) and 24 mismatching trials (4 for each of the 6 possible mismatch combinations); pseudo-randomised, no more than three consecutive matching or mismatching trials occurred sequentially.

As a second reflection of self-referential processing at a higher, conscious reflective level, participants then completed the Personal Distance Scale (PDS) (Sui & Humphreys, 2015b), wherein they moved sliders on a scale to indicate, by creating physical distance between two people, how close they were emotionally (i.e.: the smaller the value, the closer the individuals). Following several concrete examples (see Fig. 1, Part B), participants indicated, on six trials, their perception of the distance between themselves and a friend (and vice versa), themselves and a stranger (and vice versa), and their friend and a stranger (and vice versa).

Participants then completed two questionnaires: the Autism-Spectrum Quotient (AQ: Baron-Cohen et al., 2001a, b), and the UCLA Loneliness Scale (LS: Russell, 1996). Both measures boast sound psychometric qualities, with higher scores reflecting greater autistic traits and greater feelings of loneliness, respectively.

Due to time constraints in the experimental procedure, we utilized scores on the Reading the Mind in the Eyes Test (RMET: Baron-Cohen et al., 2001a, b) which had been provided by those 92 (76%) of autistic participants who had participated in our previous research (approximately 2–6 months earlier). RMET scores are stable within a 12-month period (Fernández-Abascal et al., 2013), but we thus lacked RMET data for the NA group and remaining ASC participants.

### Analysis

Our first experimental aim was to examine perceptual and higher-level SPEs and FPEs in autistic and NA participants. Due to the significant difference in age between groups, (p < 0.001) and the underrepresentation of male participants, age and sex were included as covariates in all analyses. For lower level SPEs and FPEs, the matching task yielded average accuracy and RT data for six conditions (self-matching, self-mismatching, friend-matching, friend-mismatching, stranger-matching and stranger-mismatching items). Two 3 × 2 × 2 ANOVAs (Person x Matching x Diagnosis) were conducted for accuracy and RT respectively. Where significant 3-way interactions occurred, these were explored with 6 between-subjects and 12 within-subjects post-hoc t-tests (p-values FDR-corrected). For higher-level SPE and FPEs, reflected in PDS data, we calculated average distance between self and stranger, and friend and stranger, and performed a 2 × 2 ANOVA with factors Personal Distance (Self to Stranger, Friend to Stranger) and Diagnosis. Interactions were explored with 2 between-subject and 2 within-subject post-hoc t-tests (p-values FDR-corrected).

Our second aim, addressed in correlation analysis, was to examine relationships between the SPE, the SFE, and several outcome variables: autistic traits, the theoretical construct of mentalizing (scores in the RMET; autistic group only), and a real-life social outcome (loneliness). The SPE was operationalized in six indices. Four came from the perceptual matching task: raw accuracy and RT scores for Self-Matching trials, and two continuous measures of the *extent* of the SPE in accuracy and RT, as per Williams et al. (2018), where larger scores reflected greater self-bias.^1^ Two came from the PDS: average distance between self and friend, and between self and stranger. The bias towards friends over strangers (FPE) was operationalized in three indices, two from the matching task (the difference between Friend-Matching and Stranger-Matching items in accuracy and in RT) and one from the PDS (average distance between friend and stranger).

As autism is conceptualized to exist on the extreme end of a normal distribution of autistic traits, correlations between the AQ and indices of self- and friend-bias were examined in all participants pooled. As previous studies found differences in the relationship of AQ to the SPE in autistic vs. NA populations, we then proceeded to look at this relationship and relationships with the other outcome variables in the two groups separately. Where significant relationships existed between any outcome variable and indices of the SPE or FPE, we examined whether these were specific to these stimuli or present across *all* conditions in that task. Correlations between the self-bias metrics themselves, not a focus of our analysis, can be seen in Supplementary Materials 2 and 3.

## Results

### Self and Friend Biases in Autistic and Non-autistic Participants

#### Matching Task

**Fig. 2:**
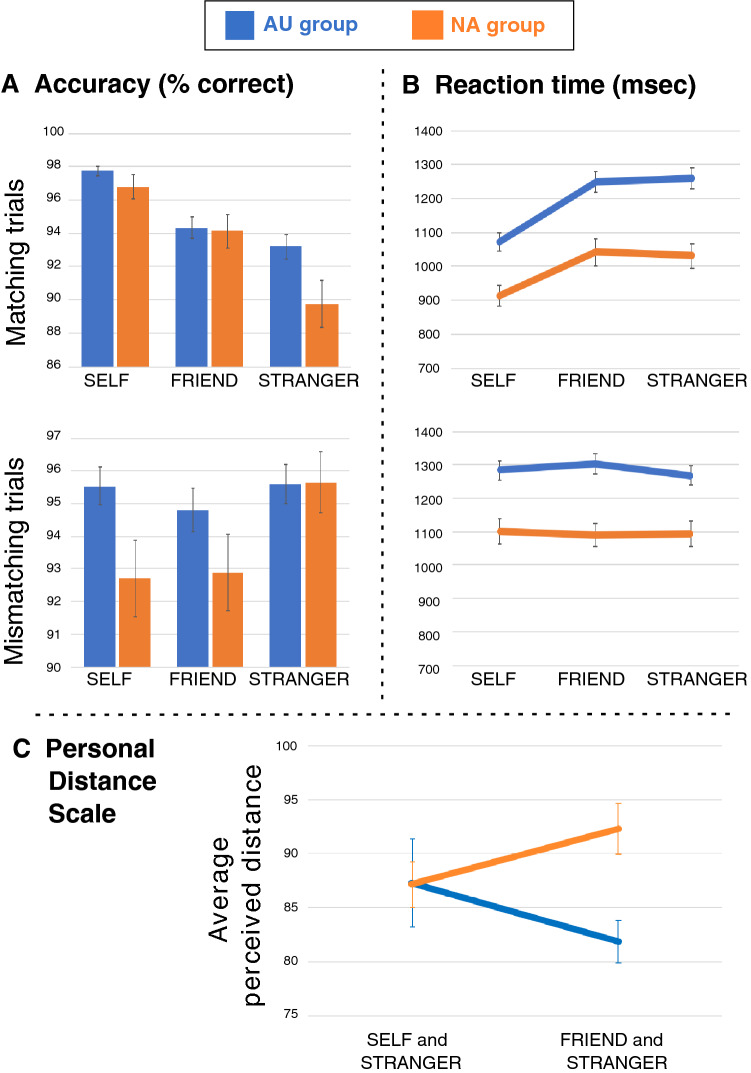
Performance of autistic (AU) and non-autistic (NA) participants in the matching task and Personal Distance Scale. Note. Averages for AU participants are displayed in blue, while those for the NA group are displayed in orange. Error bars reflect standard error. Part A depicts average accuracy (percentage correct) for each condition in matching (top) and mismatching (bottom) trials. Part B depicts average RT in milliseconds for each condition in matching (top) and mismatching (bottom) trials. Part C reflects average distances that participants perceived between themselves and a stranger, and between a stranger and a friend

As reflected in average accuracy scores (Table 1), participants performed highly accurately in the task (a main effect of Diagnosis reflected better performance overall in the autistic group: F [1, 160] = 4.08, p = 0.045). The 3 × 2 × 2 ANOVA revealed a significant interaction of Person, Matching and Diagnosis factors for accuracy (F [2, 320] = 5.76, p = 0.003). This reflected significantly greater accuracy in autistic than NA participants for Self-Mismatching items and for Stranger-Matching items—no group differences emerged for the critical Self-Matching items (Fig. 2, part A), or on the Friend-Matching items. Within-participant t-tests (see Supplementary Materials, 4) showed, however, that while autistic participants performed significantly better on Self-Matching than on Friend-Matching and Stranger-Matching trials, they were no more accurate for Friend-Matching than Stranger-Matching trials. In NA participants, an advantage of Self-Matching items was accompanied by an advantage for Friend- over Stranger-Matching items. Unlike ASC participants, NA participants also found it significantly harder to negate Self-Mismatching and Friend-Mismatching items as compared with Stranger-Mismatching items. These significant effects all survived FDR-correction.

**Table 1:** Descriptive statistics from the matching task, the Personal Distance Scale, and outcome measures

Descriptive statistics: Matching task		
Condition	ASC group	NA group
Self-Matching items	97% (3%) *1071 (303)*	97% (5%) *913 (213)*
Self-Mismatching items	95% (6%) *1281 (327)*	93% (8%) *1102 (250)*
Friend-Matching items	94% (7%) *1247 (339)*	94% (7%) *1042 (265)*
Friend-Mismatching items	95% (7%) *1302 (336)*	93% (8%) *1088 (228)*
Stranger-Matching items	93% (8%) *1256 (335)*	90% (9%) *1031 (243)*
Stranger-Mismatching items	96% (7%) *1267 (306)*	96% (6%) *1093 (253)*
Total across trials	95% (5%) *1238 (308)*	94% (5%) *1044 (227)*

Descriptive statistics: Personal Distance Scale		
Condition	ASC group	NA group
Average distance: Self and Stranger	87 (23)	87 (25)
Average distance: Friend and Stranger	81 (25)	92 (12)

Descriptive statistics: outcome measures		
Measure	ASC group	NA group
Autism Spectrum Quotient (AQ)	38.51 (7.73) Range: 40	18.82 (9.08), Range: 36
Loneliness (UCLA Loneliness Scale)	37.08 (11.84), Range: 58	22.38 (12.91), Range: 53
RMET score (average number of correct responses in the Reading the Mind in the Eyes Test)	26.44 (5.04), Range: 27	N/A

In the first part of the table (matching task), average accuracy is represented in percentages (where 100% represents perfect accuracy); average reaction times are italicized and measured in milliseconds. For the Personal Distance Scale, higher numbers reflect greater difference between two individuals (maximum possible response: 100). Throughout the table, numbers in brackets reflect standard deviation

For RT, sphericity was violated for the Person factor, so Huynh–Feldt values are reported for all effects and interactions with this factor. Main effects showed a general slowing with age (F [1, 160] = 20.87, p < 0.001), and faster performance on Matching than Mismatching trials (F [1, 160] = 20.47, p < 0.001). A significant interaction between Person and Matching (F [2, 230] = 5.41, p = 0.005) revealed the typical advantage, in both groups, for Self-items in Matching trials (see Fig. 2, Part B, and descriptive statistics in Table 1), an effect which increased with age.

#### Personal Distance Scale (PDS)

Average perceived distances between individuals are displayed in Table 1. A significant interaction in the 2 × 2 ANOVA (F [1, 153] = 11.30, p = 0.001) reflected that within-subjects, autistic participants perceived the distance between friend and stranger as significantly smaller than the distance between themselves and stranger, while NA participants placed themselves and a friend similarly far away from a stranger. Accordingly, between-subjects, the ASC group placed their friend significantly closer to the stranger (see Fig. 2, part C). These significant differences survived FDR-correction (see Supplementary Materials, 4, for full statistical notations).

### Relationships Between Self/Friend Bias and Autistic Traits, RMET Scores, and Loneliness

Participants scored in the expected range for non-autistic and autistic populations on the AQ (Baron-Cohen et al., 2014), and (autistic group only) the RMET (Baron-Cohen et al., 2015, 2001a, 2001b). No norms exist for autistic people on the UCLA Loneliness Scale (scores which range from 20–80 [Russell, 1996]), but the ASC group scored markedly higher than the non-autistic group. Some recidivism meant that not every participant completed each scale, but for those who did, our correlation analysis with metrics from the matching task is displayed in Table 2.

**Table 2:** Descriptive statistics and correlations between outcome measures and metrics from the matching task and Personal Distance Scale

	Metrics from the matching task					
	Self-Match accuracy	Self-Match RT	Self-bias acc	Self-bias RT	Friend-bias acc	Friend-bias RT
*All participants pooled*						
AQ (*n* = 158)	*r* = .10, *p* = .200	*r* = .12, *p* = .135	*r* = −.02, *p* = .790	***r* = .18, *p* = .028**	***r* = −.17, *p* = .038**	*r* = .13, *p* = .098
*Control participants*						
AQ (n = 38)	*r* = −.16, *p* = .336	*r* = .07, *p* = .690	*r* = −.05, *p* = .790	*r* = −.15, *p* = .359	*r* = .05, *p* = .767	*r* = .07, *p* = .681
Loneliness (n = 34)	*r* = .08, *p* = .640	*r* = −.09, *p* = .610	*r* = −.01, *p* = .975	*r* = .04, *p* = .815	*r* = .21, *p* = .235	*r* = .28, *p* = .106
*Autistic participants*						
AQ (n = 120)	*r* = .14, *p* = .142	*r* = −.09, *p* = .353	*r* = .03, *p* = .723	*r* = .14*,p* = .126	*r* = −.07, *p* = .475	*r* = .10, *p* = .302
Loneliness (n = 119)	*r* = −.09, *p* = .323	***r* = .24, *p* = .008**	*r* = .09, *p* = .324	*r* = .06, *p* = .527	*r* = −.04, *p* = .658	*r* = .09, *p* = .348
RMET (n = 78)	*r* = .16, *p* = .160	*r* = −.03, *p* = .778	*r* = .01, *p* = .987	*r* = .13, *p* = .260	*r* = −.13, *p* = .268	*r* = −.11, *p* = .340

	Metrics from Personal Distance Scale		
	Self-Friend distance	Self-Stranger distance	Friend-Stranger distance
*All participants pooled* AQ (*n* = 158)	*r* = .10, *p* = .206	*r* = −.06, *p* = .495	***r* = −.16, *p* = .042**
Loneliness (n = 153)	***r* = .22, *p* = .007**	*r* = −.11, *p* = .179	***r* = −.21, *p* = .01**
*Control participants* AQ (n = 38)	*r* = .91, *p* = .587	*r* = −.08, *p* = .620	*r* = −.12, *p* = .459
Loneliness (n = 34)	*r* = −.03, *p* = .881	*r* = .05, *p* = .780	*r* = −.16, *p* = .352
*Autistic participants*			
AQ (n = 120)	*r* = .04*, p* = .706	*r* = −.08, *p* = .378	*r* = −.02, *p* = .808
Loneliness (n = 119)	***r* = .32, *p* < .001**	***r* = −.20, p = .034**	*r* = −.14,* p* = .122
RMET (n = 78)	*r* = −.09, *p* = .424	*r* = .11, *p* = .339	*r* = .00, *p* = .988

Significant relationships are highlighted in bold font. Labels for metrics from the matching task and Personal Distance Scale reflect: Self-Match accuracy (average accuracy in Self-Matching trials); Self-Match RT (average RT in Self-Matching trials); Self-bias acc. (the extent of the self-bias calculated from accuracy scores); Self-bias RT (the extent of the self-bias calculated from RT data); Friend-bias acc. (the extent of the friend-bias calculated from accuracy scores); Friend-bias RT (the extent of the friend-bias calculated from RT data). Acronyms for outcome measures include AQ (scores on the Autism-Spectrum Quotient); RMET (scores on the Reading the Mind in the Eyes Test); Loneliness (scores on the UCLA Loneliness Scale)

Only in all participants pooled did any relationships emerge between autistic traits and indices of the SPE and FPE. A greater degree of self-bias in RT was associated with higher autistic traits; conversely, as autistic traits increased, the friend-bias in accuracy decreased.

Though RMET scores did not correlate with AQ, loneliness or any of our self- or friend-bias metrics, loneliness correlated with autistic traits in both groups (NA participants: r = 0.44, p = 0.009; autistic participants: r = 0.19, p = 0.039). Interestingly, a highly significant relationship in the autistic group suggested that lonelier autistic people tended to have longer reaction times for Self-Matching Items. Closer scrutiny of the specificity of this relationship revealed, however, that loneliness in autistic people was associated with higher RTs across *all* trials (r = 0.25, p = 0.005).

Analysis of indices from the PDS revealed a positive association between loneliness and distance between friend and self in all participants pooled (and in the ASC group alone). With reference to the FPE, two negative correlations showed that as autistic traits and loneliness increased, the distance between friend and stranger decreased. In the ASC group, furthermore, lonelier participants perceived the self and stranger to be closer together.

**Fig. 3:**
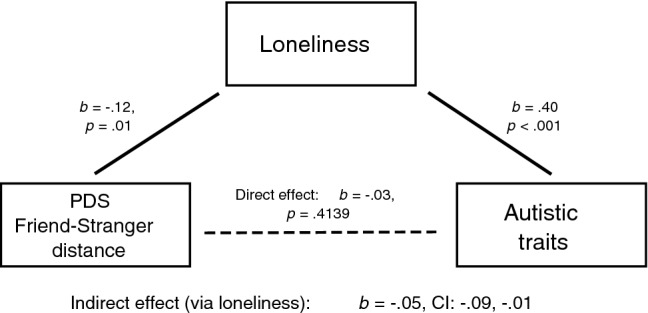
Post-hoc mediation analysis of relationships between Friend-Stranger distance, loneliness and autistic traits. Note. Figure displays coefficients and p values for relationships between Friend-Stranger distance (Personal Distance Scale), loneliness and autistic traits

Given the observed relationships between autistic traits, loneliness, and perceived distance between friend and stranger, we conducted a post-hoc mediation analysis with PROCESS (Hayes, 2017) to examine whether, in all participants, the inverse relationship between autistic traits and friend-stranger distance was direct or mediated by loneliness. The relationship was indeed fully mediated by loneliness (see Fig. 3).

## Discussion

With conflicting findings around SPE in autism, the aims of this experiment were twofold. Firstly, we sought to investigate group differences between autistic and non-autistic participants in the self-prioritization effect (SPE), and the previously uninvestigated friend-prioritisation effect (FPE), across both lower-level perceptual and higher-level tasks. Secondly, motivated by the connection between self-representation, other-representation and social functioning, we explored relationships between these effects and measures of autistic traits, mentalizing ability, and loneliness as an outcome of social dysfunction. With reference to the primary goal, post-hoc exploration of interactions in our tasks suggested subtle group differences in lower- and higher-level representation of self and others.

### Self- and Friend-Prioritization in Autistic and Non-autistic Participants

The typical prioritization of self-relevant information is robust across paradigms (Sui & Humphreys, 2015a), but called into question with contradictory findings in autism. The mnemonic SPE has been found absent or reduced in autistic populations (Burrows et al., 2017; Grisdale et al., 2014; Henderson et al., 2009; Lombardo et al., 2007), but previous studies of SPE at the perceptual level (Williams et al., 2018) reported evidence of self-prioritisation much like our own findings around accuracy and RT in the perceptual task. This might be a property of task differences, where unlike tasks which require consideration of the self as an object of thought, our virtually identical speeded matching tasks discourage or disallow deeper processing of the self as a second-order representation (Williams et al., 2018). Likewise, recent work suggests that the abstractness of paradigms such as the self-ownership paradigm is confounding, such that mnemonic advantages for self-owned items may depend on grounding the notion of self-ownership in concrete actions (Wuyun et al., 2020).

Whilst our data cannot decisively dispute or support these ideas, subtle differences in the positioning and prioritizing of the self-concept in relation to familiar and unfamiliar others refute the notion that self-representation is entirely typical. Our late-diagnosed autistic participants found it significantly easier to negate Self-Mismatching items than did NA participants. Comparing within-subjects, NA participants had more difficulty negating Self- and Friend-Mismatching trials, suggesting that they found it more difficult to ‘unhook’ themselves from Self-Mismatching trials and extended the same privilege to Friend trials. The ‘stuckness’ of NA participants in mismatching trials relates to the highjacking of attentional resources by self-relevant stimuli (Röer & Cowan, 2020). At the neural level, the same pattern of behaviour was associated with activation of left posterior superior temporal sulcus (involved in attentional orientation to socially salient stimuli) and the strength of its connection with ventromedial prefrontal cortex, which is strongly implicated in self-bias effects (Jie Sui, et al., 2013a, 2013b). The higher accuracy and greater ease of processing shown by autistic participants in all mismatching trials, and the group difference between autistic and NA participants in ease of negating self-mismatching trials, may perhaps imply reduced specificity and reduced attentional capture by self-relevant stimuli in autistic participants. This interpretation is reminiscent of a common phenomenon in autistic children, the lack of orientating response to own name (Lombardo & Baron-Cohen, 2010), which is believed to be highly relevant to differences in joint attention and later social-cognitive development (Parise et al., 2010). A recent investigation in autistic adults linked a lack of preferential neural response to own name to diminished activity in the right temporoparietal junction (Nijhof et al., 2018), an area previously implicated as a “low-level neurocognitive mechanism of self-other distinction” (p. 136).

In addition to being more ‘stuck’ on Friend-Mismatching than Stranger-Mismatching trials, within-subject tests showed that NA participants made more accurate responses to Friend-Matching trials than to Stranger-Matching trials. Other studies have shown that prioritisation of familiar others often aligns across higher- and lower-level tasks (Sui & Humphreys, 2017), and this also appears to be the case in the present dataset, where the diminishment of lower-level FPE in autistic participants also seems inherent in the placement of the friend and stranger concepts in our higher-level processing task, the PDS. Friend-prioritization was evident in NA participants’ placing of the stranger concept similarly far away from themselves *and* their friend. Autistic people, in contrast, placed their friend significantly closer to the stranger than they did their selves. The only group difference lay in the closeness of the friend to the stranger.

It is important to recognize that the FPE is strongly related to the SPE, so perturbations in the FPE are also reflective of self-prioritisation. The FPE reflects a form of in-group bias, which is itself believed to be rooted in self-prioritisation: in-groups accrue preferential significance through their association with the self-concept (Enock et al., 2020; Moradi et al., 2020; van Veelen et al., 2016). Less is known about in-group bias in autistic people, but it has been seen to be reduced in adults with higher autistic traits (Bertschy et al., 2020), and this was indeed related to reduced self-categorization. These authors have emphasized that the self-concept, and processing of the self in relation to others, is important for the formation of social identity and identification with in-groups, and that these processes are themselves important for relationships, mental health and wellbeing (Bertschy et al., 2020; Skorich et al., 2017). Whether an in-group bias exists in autism, its relation to the self-concept and putative downstream consequence on relationships and broader wellbeing, is an intriguing target for future research.

### Relationships Between Self- and Friend-Representation, Autistic Traits, Mentalizing and Loneliness

The conceptual relationship between autistic traits and ASC (Baron-Cohen et al., 2001a, b) implies that a reduction of SPE in autistic people would be accompanied by increasing autistic traits, a hypothesis supported by some studies (Grisdale et al, 2014; Henderson et al., 2009; Lombardo et al., 2007) but not others (Lind et al., 2020; Williams et al., 2018). Likewise, most of our SPE indices were unrelated to AQ—but we did observe a relationship, in all participants, between greater self-bias in reaction time (where higher scores reflected preferential processing of self-matching over friend- and stranger-matching items) and higher autistic traits. This is intriguing in its resemblance with findings from Lombardo et al., who found the slope of the relationship between autistic traits and SPE was dependent on diagnostic status: while reduced SPE were associated with higher autistic traits in autistic individuals, in non-autistic participants autistic traits increased with greater self-prioritization.

Comparison between studies is challenged by measurement, task and sample invariance, but recent investigations may reconcile some discrepancies. Gillespie-Smith and colleagues (2018) found that autistic children with fewer autistic traits had a *more pronounced SPE* than NA children (which might reflect difficulty disengaging from the self), whereas autistic children classed as ‘severely affected’ had markedly reduced SPE. If differences in self-representation do not diverge categorically by diagnosis per se, as also implied in Burrows et al. (2017), inconsistent relationships may reflect chance recruitment of participants with different autistic presentations or levels of autistic traits. As noted in the resemblance with findings from NA participants in Lombardo et al., our late-diagnosed participants might be more comparable with the NA than the heterogenous autistic population, a hypothesis inviting future investigation.

The same speculation may be pertinent to the relationship between SPE and mentalizing, which share neural substrates in the brain areas underpinning self- and other-representation. Like previous studies (Henderson et al., 2009; Williams et al., 2018), we failed to find any relationships between self- or friend-prioritization effects and the RMET, which was further unrelated to autistic traits or loneliness. Noting its dissociation from other mentalizing tasks, some theorists argue that the RMET actually reflects emotion recognition (Livingston, et al., 2019a, 2019b), which may explain why it does not reflect the overlap between self- and other-processing.

Mentalizing tasks are, generally, weakly linked to real-world social impairments (Bottema-Beutel et al., 2019), hence our interest in loneliness as a potential correlate of self-processing differences. Relationships between autistic traits and loneliness are well-established (Reed et al., 2016; Stice & Lavner, 2019), but the association between loneliness and slower processing of Self-Matching items was non-specific; loneliness was linked with slower processing globally, perhaps due to its relationship with depression (Hedley et al., 2018), which also impedes cognition (Shura et al., 2017). In the PDS, loneliness predicted the distance between self and friend for autistic and all participants pooled, and between self and stranger for autistic participants alone. With all participants pooled, both loneliness and autistic traits predicted the distance between friend and stranger, and post-hoc mediation analysis showed that loneliness fully mediated (i.e. explained) the tendency of participants with higher autistic traits to perceive friends and strangers closer together. Our suggestion of directionality is, of course, speculative in these analyses, but they highlight loneliness as a variable which might also help to clarify some of the inconsistencies around relationships between autistic traits, sociocommunicative processes and levels of self-processing. Given the involvement of the self-concept in facets of cognitive and social functioning of relevance for relationships and wellness (Bertschy et al., 2020; Nijhof & Bird, 2019; Skorich et al., 2017) and its potential to derail development in these domains, these analyses also invite us, again, to consider far-upstream roles of differences in the self-concept and self-processing which may be under-appreciated in the everyday difficulties facing autistic people.

### Limitations and Future Directions

The study raises several novel points and directions for future research. The patterns in our data suggest that differences in self-representation in autistic people may be subtle and possibly obfuscated by sample characteristics. While interactions in RT and accuracy in the matching task suggested a normalized SPE, closer examination of post-hoc tests and the inclusion of another measure of self-referential processing suggested reduced specificity of the self-concept and differences in its positioning in relation to others. One reflection of this is the FPE, with its connection with in-group bias and self-relevance (Enock et al., 2020; Moradi et al., 2020; van Veelen et al., 2016); this presents a novel and intriguing avenue for future research in autism. The self is a multidimensional concept that permeates cognition at many levels, with apparently ‘lower level’ processes manifesting in higher level phenomena (Sui & Gu, 2017). Clarification may as such be found in expanding the paradigms used to measure it within a single study; the same argument has been made regarding mentalizing (Bottema-Beutel et al., 2019), and would be relevant for future exploration of the connection between self- and other-processing.

There were several limitations on which future studies might improve. Our control group was likely underpowered, and uncontrolled variables could have contributed to within- and between-group variance and might partly account for inconsistencies to the literature around SPE in autism. Alongside loneliness, we noted depression as a potential confound of group comparisons and a source of variance, given its commonality in autism (Hudson et al., 2019) and the fact that negative mood is associated with diminished SPE (Fan et al., 2016; Sui et al., 2016). We were unable to examine sex as a variable of interest due to underrepresentation of male participants in both groups. As the reported tasks were embedded within a larger study ostensibly about mental health, this may reflect the typically higher take-up of online mental health research by women (Aerny-Perreten et al., 2015; Choi et al., 2017). No main effects of sex were observed (as a covariate), but this is unsurprising. In that sex has a marked influence on experiences of loneliness and friendship (Hall, 2011; Stokes & Levin, 1986) and even moderates brain activity during self-representation in autistic and non-autistic men and women (Lai et al., 2019), this variable may be important to factor into future analyses.

Given the over-representation of women in our sample, it is likely no coincidence that the majority of participants were diagnosed in adulthood. The profile of the late-diagnosed autistic adult dovetails, to some degree, with the profile of autistic women (Lawson, 2019; Lehnhardt et al., 2016). The prevalence of autism in girls and women has recently been placed at approximately 3.3 females to every male (Loomes et al., 2017), but confidence in any estimates is challenged by known biases in assessment tools, lay and clinician perceptions of autism, all of which mean that autistic girls are diagnosed later, if before adulthood (Lai & Szatmari, 2020; Lockwood Estrin et al., 2020). A bimodal distribution of prevalence in autistic females reflects that those with more obvious presentation are often diagnosed in childhood, whereas our sample may be more reflective of those more verbal women with IQ in the high-average range who are diagnosed as adults (Van Wijngaarden-Cremers et al., 2014). In that approximately half of our sample were employed and the majority qualified to degree level, we might speculate that they possessed above-average ability to camouflage and compensate for their difficulties, and consequently may have possessed slightly superior executive function, verbal IQ and expressive language than others within the autistic population (Corbett et al., 2021; Hull et al., 2020; Livingston, et al., 2019a, 2019b). While the cognitive profile of our sample might differ from that of autistic men and women diagnosed in childhood, it is inarguable that they are unrepresentative of autistic people with intellectual disability. In that these individuals constitute possibly 40% of the autistic population (Autistica, 2021) and are conspicuously absent from investigations of self-referential processing (Wuyun et al., 2020, is one known exception), differences in self-representation and their sociocognitive impact on this group are, as such, strongly deserving of research attention. As the SPE may be strongly dependent on variation within the autistic community (Burrows et al., 2017; Gillespie-Smith et al., 2018), in-depth analysis within large samples may be fruitful to identifying other moderators of the SPE, alongside sex, that might be missed in more simple categorical comparisons between autistic and non-autistic people.

Other barriers to the generalizability of our findings include that they are culture-bound, and effects of culture may require disentangling from effects of autism. While our findings in accuracy data from the matching task suggested reduced specificity of the self-concept in autism, Wuyun et al. (2020) found similar equivalence in the way autistic children processed self- and close other-owned objects; they, however, queried whether this reflected a more “interdependent self” in Chinese culture. Secondly, concerns have been raised over the use and representativeness of undergraduate samples such as our small NA group (DeRight & Jorgensen, 2015; Hanel & Vione, 2016), and the reproducibility of such findings (Peterson & Merunka, 2014). Our online study likely excluded autistic *and* non-autistic individuals with poorer computer literacy and those for whom this technology was inaccessible.

## Conclusion

While group differences in self-representation were not immediately apparent in main effects and interactions in a lower-level matching task, post-hoc tests and the inclusion of a higher-level processing task suggested differences did indeed exist in how our NA participants and late-diagnosed autistic participants represented the self-concept and its positioning in relation to others. We suggest these subtle differences and relationships between self-representation and broader sociocognitive abilities may be obfuscated by sample heterogeneity and several potential confounding variables. In that differences in the self-concept and its relation to others have far-reaching implications on social processes and relationships, further multi-level exploration of the self in autism, and real-world downstream implications of these differences, is warranted.

## Supplementary Information

Below is the link to the electronic supplementary material.

Supplementary file1 (DOCX 28 KB)

## Footnotes

1 To calculate the extent of self-bias in accuracy, average accuracy for Self-Matching items was subtracted from the averaged averages of Friend-Matching and Stranger-Matching items. For the extent of self-bias in RT, each participant’s average RT for Friend-Matching and Stranger-Matching items were themselves averaged and subtracted from their average RT to Self-Matching items.
