# Supplementary material for: Levels of Self-representation and Their Sociocognitive Correlates in Late-Diagnosed Autistic Adults
Source: J Autism Dev Disord. 2021 Aug 30;52(7):3246–59. doi: 10.1007/s10803-021-05251-x (PMC9213305; doi:10.1007/s10803-021-05251-x)
Supplement: Supplementary file 1 — Supplementary file1 (DOCX 28 KB) [file 10803_2021_5251_MOESM1_ESM.docx]

**Supplementary materials**

Table of contents:

1. Pilot study
2. Correlations between self/friend-bias metrics in the matching task
3. Correlations between self/friend-bias metrics in the PDS and matching task
4. Post-hoc t-tests for interactions seen in group comparisons

Supplementary item 1: Pilot study

The online matching task was piloted on 21 non-autistic (NA) volunteers who did not participate in the real study. We did not apply counterbalancing of shape-label pairings in the pilot, so in this version of the task, ‘square’ was paired with ‘yourself’, ‘circle’ with ‘friend’, and ‘triangle’ with ‘stranger’.

To make this task as clear as possible for an online audience, explicit instructions were provided with two self-paced associative learning phases. In the first, participants viewed the three pairings of shapes and person-labels; in the second, they viewed one correct and incorrect presentation of two word-shape pairings with the appropriate responses indicated (‘This time the word is with the shape it belongs with, so you’d press **M** (it **M**atches)’; ‘This time the word is NOT with the shape it belongs with. Remember, ___ belongs with ____. So you’d press **N** (**N**o, it does **NOT** match)’). With the shape treated as a constant, ‘mismatches’ were defined by whether or not the **labels** attached to the shapes were correct: mismatches were therefore instances where the label paired with the shape was not the label linked to that shape during the learning phase. This was followed by two rounds of practice trials, interspersed with a reminder of the correct pairings of labels and shapes. The first 12 practice trials were self-paced: participants saw a fixation cross for 2000ms, followed by the word and person-label which stayed on the screen indefinitely until they responded. Responses prompted a feedback screen (1000ms) where correct responses received a green thumbs up, incorrect responses and misses a red thumbs down. The second round of practice trials exposed participants to 12 trials at real speed. After another reminder of the shape-label pairings, they completed three experimental blocks (approximately 3.6 minutes each), interspersed with self-timed breaks and reminders of the shape-label pairings.

With an average accuracy of 91% for all trials (SD: 9%), most participants in this pilot study performed highly accurately (the lowest had an overall accuracy of 61%). A 3x2 analysis of variance (ANOVA) was performed with the levels Person (3: Self, Friend or Stranger) and Matching (2 levels: Match or Mismatch). No effects emerged in accuracy data, but in reaction times (RT), a significant effect of Person (*F* (2, 38) = 8.865, *p* = .001) reflected fastest response to Self items, followed by Friend items, followed by Stranger items; a significant effect of Matching (*F* (1, 19) = 9.049, *p* = .007) reflected that participants were faster to confirm matches than negate mismatches; and an interaction between Person and Matching (*F* (2, 38) = 5.883, *p* = .006) where the self-bias effect emerged in markedly faster responses to Self-Matching items above any other trial type.

Supplementary item 2: Correlations between self/friend-bias metrics in the matching task

Black text here reflects results from all participants pooled.

Blue text reflects results from autistic participants only.

Orange text reflects results from NA participants only.

|  | Matching task:  Self-Match accuracy | Matching task:  Self-Match RT | Matching task:  Self-bias acc. | Matching task:  Self-bias RT | Matching task:  Friend-bias acc. | Matching task:  Friend-bias RT |
| --- | --- | --- | --- | --- | --- | --- |
| Matching task:  Self-Match accuracy |  |  |  |  |  |  |
| Matching task:  Self-Match RT | *r* = -.17, *p* = .03  *r* = -.20, *p* = .03  *r* = -.259, *p* = .09 |  |  |  |  |  |
| Matching task:  Self-bias acc. | *r* = .18, *p* = .02  *r* = .19, *p* = .041  *r* = .203, *p* = .187 | *r* = -.064, *p* = .416  *r* = -.03, *p* = .744  *r* = .059, *p* = .704 |  |  |  |  |
| Matching task:  Self-bias RT | *r* = .27, *p* = .001  *r* = .28, *p* = .002  *r* = .248, *p* = .105 | *r* = -.05, *p* = .511  *r* = -.12, *p* = .189  *r* = -.137, *p* = .376 | *r* = .328, *p* < .001  *r* = .40, *p* < .001  *r* = .125, *p* = .420 |  |  |  |
| Matching task:  Friend-bias acc. | *r* = -.038, *p* = .631  *r* = -.053, *p* = .565  *r* = .052, *p* = .737 | *r* = -.111, *p* = .154  *r* = -.029, *p* = .755  *r* = -.233, *p* = .128 | *r* = .236, *p* = .002  *r* = .150, *p* = .100  *r* = 452, *p* = .002 | *r* = -.014*, p* = .854  *r* = .040, *p* = .663  *r* = -.084, *p* = .590 |  |  |
| Matching task:  Friend-bias RT | *r* = -.034, *p* = .663  *r* = -.115, *p* = 210  *r* = .105, *p* = .497 | *r* = .132, *p* = .091  *r* = -.127, *p* = .164  *r* = -.263, *p* = .084 | *r* = -.018*, p* = .822  *r* = .037, *p* = .690  *r* = -.161, *p* = .295 | *r* = .180, *p* = .02  *r* = .182, *p* = .046  *r* = .140, *p* = .365 | *r* = .235, *p* = .002  *r* = .255, *p* = .005  *r* = .246, *p* = .107 |  |

Supplementary item 3: Correlations between self/friend-bias metrics in the PDS and matching task

Though not the focus of this investigation, we furthermore checked to ensure expected relationships were present between the four PDS variables from this conscious measure of self-perception and the metrics of self-bias from the more autonomous matching task. From the matching task, for all participants pooled, longer RT in Self-Matching trials was significantly associated with greater distance perceived between Self and Friend (*r* = .23, *p* =.004); conversely, shorter RT were associated with greater perceived distance between Self and Stranger (*r* = -.20, *p* = .012). There were no correlations between any of the PDS variables and accuracy to Self-Match items, or the extent of self-bias in accuracy or self-bias in RT. For autistic participants alone, the same two correlations were significant (*p* < .025) in the same directions; for the NA group, these were non-significant.

As before, black text reflects correlations with all participants pooled; blue text reflects correlations computed with autistic participants only; and orange text reflects results from NA participants only.

|  | PDS Self-Friend distance | PDS Self-Stranger distance | PDS Friend-Stranger distance |
| --- | --- | --- | --- |
| PDS Self-Friend distance |  |  |  |
| PDS Self-Stranger distance | *r* = -.339, *p* < .001  *r* = -.122, *p* = .187  *r* = -.259, *p* = .09 |  |  |
| PDS Friend-Stranger distance | *r* = -.081, *p* = .316  *r* = -.087, *p* = .347  *r* = .203, *p* = .187 | *r* = .650, *p* < .001  *r* = .795, *p* < .001  *r* = .059, *p* = .704 |  |
| Matching task:  Self-Match accuracy | *r* = -.019, *p* = .813  *r* = -.066, *p* = .478  *r* = .203, *p* = .187 | *r* = .052, *p* = .516  *r* = .076, *p* = .408  *r* = .059, *p* = .704 | *r* = -.037, *p* = .643  *r* = -.020, *p* = .825  *r* = -.263, *p* = .084 |
| Matching task:  Self-Match RT | *r* = .230, *p* = .004  *r* = .218, *p* = .017  *r* = .203, *p* = .187 | *r* = -.198, *p* = .012  *r* = -.205, *p* = .024  *r* = .059, *p* = .704 | *r* = -.165, *p* = .039  *r* = -.171, *p* = .063  *r* = -.263, *p* = .084 |
| Matching task:  Self-bias acc. | *r* = .094, *p* = .243  *r* = .156, *p* = .091  *r* = .248, *p* = .105 | *r* = -.134, *p* = .094  *r* = -.241, *p* = .008  *r* = -.137, *p* = .376 | *r* = -.070, *p* = .386  *r* = -.103, *p* = .266  *r* = .125, *p* = .420 |
| Matching task:  Self-bias RT | *r* = .099, *p* = .218  *r* = .036, *p* = .697  *r* = .052, *p* = .737 | *r* = -.143, *p* = .073  *r* = -.106, *p* = .249  *r* = -.233, *p* = .128 | *r* = -.069, *p* = .391  *r* = -.061, *p* = .512  *r* = 452, *p* = .002 |
| Matching task:  Friend-bias acc. | *r* = -.070, *p* = .386  *r* = .092, *p* = .318  *r* = .105, *p* = .497 | *r* = -.073, *p* = .360  *r* = -.223, *p* = .014  *r* = -.263, *p* = .084 | *r* = -.101, *p* = .210  *r* = -.179 *p* = .051  *r* = -.161, *p* = .295 |
| Matching task:  Friend-bias RT | *r* = .007, *p* = .930  *r* = .042, *p* = .653  *r* = .203, *p* = .187 | *r* = -.084, *p* = .296  *r* = -.110, *p* = .232  *r* = -.263, *p* = .084 | *r* = -.041, *p* = .613  *r* = -.014, *p* = .876  *r* = -.263, *p* = .084 |

Supplementary item 4: Post-hoc t-tests for interactions seen in group comparisons

Where significant effects were seen in ANOVA analyses, these were explored in post-hoc within- and between-subject t-tests as indicated. The statistical notations for these are summarized in the main text and reported below in full.

***Accuracy: matching task***

Interaction: Person × Matching × Diagnosis: *F* (2, 320) = 5.76, *p* = .003

**Between-subjects t-tests:**

Self-Matching items: NS

Self-Mismatching items: *t* [163] = 2.37, *p* = .019 (FDR correction: *p* = .043)

Friend-Matching items: NS

Friend-Mismatching items: NS

Stranger-Matching items: *t* [163] = 2.28, *p* = .024 (FDR correction: *p* = .048)

Stranger-Mismatching items: NS

**Within-subjects t-tests: AU group**

Self-Matching items vs. Friend-Matching items: *t* [120] = 5.40, *p* < .001 (FDR correction: *p* < .001)

Self-Matching items vs. Stranger-Matching items: *t* [120] = 6.31, *p* < .001 (FDR correction: *p* < .001)

Friend-Matching items vs. Stranger-Matching items: NS

Self-Mismatching items vs. Friend-Mismatching items: NS

Self-Matching items vs. Stranger-Mismatching items: NS

Friend-Mismatching items vs. Stranger-Mismatching items: NS

**Within-subjects t-tests: NA group**

Self-Matching items vs. Friend-Matching items: *t* [43] = 2.98, *p* = .005 (FDR correction: *p* = .013)

Self-Matching items vs. Stranger-Matching items: *t* [43] = 5.26, *p* < .001 (FDR correction: *p* < .001)

Friend-Matching items vs. Stranger-Matching items: *t* [43] = 3.96, *p* < .001 (FDR correction: *p* < .001)

Self-Mismatching items vs. Friend-Mismatching items: NS

Self-Mismatching items vs. Stranger-Mismatching items: *t* [43] = 3.14, *p* = .003 (FDR correction: *p* = .011)

Friend-Mismatching items vs. Stranger-Mismatching items: *t* [43] = 2.93, *p* = .005 (FDR correction: *p* = .013)

***Personal Distance Scale***

Interaction: Personal distance x Diagnosis: *F* (1, 153) = 11.30, *p* = .001

**Between-subjects t-tests:**

Distance between Self and Stranger: NS

Distance between Friend and Stranger: *t* [155] = 2.43, *p* = .016 (FDR correction: *p* = .032)

**Within-subjects t-tests: AU group**

Distance between Self and Stranger vs. distance between Friend and Stranger: *t* [118] = 3.65, *p* < .001 (FDR correction: *p* < .001)

**Within-subjects t-tests: NA group**

Distance between Self and Stranger vs. distance between Friend and Stranger: NS
